# Author Correction: DNA methylation predicts age and provides insight into exceptional longevity of bats

**DOI:** 10.1038/s41467-021-23129-5

**Published:** 2021-05-05

**Authors:** Gerald S. Wilkinson, Danielle M. Adams, Amin Haghani, Ake T. Lu, Joseph Zoller, Charles E. Breeze, Bryan D. Arnold, Hope C. Ball, Gerald G. Carter, Lisa Noelle Cooper, Dina K. N. Dechmann, Paolo Devanna, Nicolas J. Fasel, Alexander V. Galazyuk, Linus Günther, Edward Hurme, Gareth Jones, Mirjam Knörnschild, Ella Z. Lattenkamp, Caesar Z. Li, Frieder Mayer, Josephine A. Reinhardt, Rodrigo A. Medellin, Martina Nagy, Brian Pope, Megan L. Power, Roger D. Ransome, Emma C. Teeling, Sonja C. Vernes, Daniel Zamora-Mejías, Joshua Zhang, Paul A. Faure, Lucas J. Greville, Steve Horvath, L. Gerardo Herrera M., José J. Flores-Martínez

**Affiliations:** 1 Department of Biology, University of Maryland, College Park, MD USA; 2 Department of Human Genetics, David Geffen School of Medicine, University of California, Los Angeles, CA USA; 3 Department of Biostatistics, Fielding School of Public Health, University of California, Los Angeles, CA USA; 4 Altius Institute for Biomedical Sciences, Seattle, WA USA; 5 Department of Biology, Illinois College, Jacksonville, IL USA; 6 Department of Anatomy and Neurobiology, Northeast Ohio Medical University, Rootstown, OH USA; 7 Department of Evolution, Ecology and Organismal Biology, The Ohio State University, Columbus, OH USA; 8 Department of Migration, Max Planck Institute of Animal Behavior, Radolfzell, Germany; 9 Department of Biology, University of Konstanz, Konstanz, Germany; 10 Smithsonian Tropical Research Institute, Panama, FL USA; 11 Neurogenetics of Vocal Communication Group, Max Planck Institute for Psycholinguistics, Nijmegen, The Netherlands; 12 Department of Ecology and Evolution, University of Lausanne, Lausanne, Switzerland; 13 Museum für Naturkunde, Leibniz-Institute for Evolution and Biodiversity Science, Berlin, Germany; 14 School of Biological Sciences, University of Bristol, Bristol, UK; 15 Department Biology II, Ludwig Maximilians University Munich, München, Martinsried Germany; 16 Department of Biology, State University of New York, Geneseo, NY USA; 17 Instituto de Ecología, Universidad Nacional Autónoma de México, Ciudad Universitaria, Mexico City, Mexico; 18 Lubee Bat Conservancy, Gainesville, FL USA; 19 School of Biology and Environmental Science, University College Dublin, Belfield, Dublin 4 Ireland; 20 Donders Institute for Brain, Cognition and Behaviour, Nijmegen, The Netherlands; 21 School of Biology, The University of St Andrews, Fife, UK; 22 Department of Psychology, Neuroscience and Behaviour, McMaster University, Hamilton, ON Canada; 23 Estación de Biología de Chamela, Instituto de Biología, Universidad Nacional Autónoma de México, 48980 San Patricio, Mexico; 24 Departamento de Zoología, Instituto de Biología, Universidad Nacional Autónoma de México, 04510 Ciudad de México, Mexico

**Keywords:** Epigenomics, Methylation analysis, Ageing

Correction to: *Nature Communications* 10.1038/s41467-021-21900-2, published online 12 March 2021.

The original version of this Article omitted from the author list the 34th author L. Gerardo Herrera M., who is from the ‘Estación de Biología de Chamela, Instituto de Biología, Universidad Nacional Autónoma de México, 48980 San Patricio, Mexico’ and the 35th author José J. Flores-Martínez, who is from the ‘Departamento de Zoología, Instituto de Biología, Universidad Nacional Autónoma de México, 04510 Ciudad de México, Mexico’. Additionally, the following was added to the Author Contributions: ‘L.G.H.M., J.J.F.M., M.N., B.P., M.L.P., R.D.R., E.C.T., S.C.V., G.S.W., and D.Z. provided or prepared samples’ in place of ‘M.N., B.P., M.L.P., R.D.R., E.C.T., S.C.V., G.S.W., and D.Z. provided or prepared samples.’

The original version of the Supplementary Information associated with this Article contained an error in p. 4, which incorrectly read ‘Bat capture and handling were conducted under permits #7668-15 and 2492-17 from Dirección General de Vida Silvestre, and permits #17-16 and 21-17 from Secretaría de Gobernación.’ The correct version states ‘#7668-15, 2492-17 and 5409-18’ in place of ‘#7668-15, 2492-17’ and ‘#17-16, 21-17 and 20-18’ in place of ‘#17-16 and 21-17.’ The HTML has been updated to include a corrected version of the Supplementary Information.

The original version of this Article contained an error in the email address of the corresponding author Steve Horvath, which was missing the terminal edu and should be shorvath@mednet.ucla.edu

These have been corrected in both the PDF and HTML versions of the Article.

## Supplementary Information

Supplementary Information

