# Author Correction: DNA methylation predicts age and provides insight into exceptional longevity of bats

**DOI:** 10.1038/s41467-022-33102-5

**Published:** 2022-09-07

**Authors:** Gerald S. Wilkinson, Danielle M. Adams, Amin Haghani, Ake T. Lu, Joseph Zoller, Charles E. Breeze, Bryan D. Arnold, Hope C. Ball, Gerald G. Carter, Lisa Noelle Cooper, Dina K. N. Dechmann, Paolo Devanna, Nicolas J. Fasel, Alexander V. Galazyuk, Linus Günther, Edward Hurme, Gareth Jones, Mirjam Knörnschild, Ella Z. Lattenkamp, Caesar Z. Li, Frieder Mayer, Josephine A. Reinhardt, Rodrigo A. Medellin, Martina Nagy, Brian Pope, Megan L. Power, Roger D. Ransome, Emma C. Teeling, Sonja C. Vernes, Daniel Zamora-Mejías, Joshua Zhang, Paul A. Faure, Lucas J. Greville, L. Gerardo Herrera M., José J. Flores-Martínez, Steve Horvath

**Affiliations:** 1 Department of Biology, University of Maryland, College Park, MD USA; 2 Department of Human Genetics, David Geffen School of Medicine, University of California, Los Angeles, CA USA; 3 Department of Biostatistics, Fielding School of Public Health, University of California, Los Angeles, CA USA; 4 Altius Institute for Biomedical Sciences, Seattle, WA USA; 5 Department of Biology, Illinois College, Jacksonville, IL USA; 6 Department of Anatomy and Neurobiology, Northeast Ohio Medical University, Rootstown, OH USA; 7 Department of Evolution, Ecology and Organismal Biology, The Ohio State University, Columbus, OH USA; 8 Department of Migration, Max Planck Institute of Animal Behavior, Radolfzell, Germany; 9 Department of Biology, University of Konstanz, Konstanz, Germany; 10 Smithsonian Tropical Research Institute, Panama, FL USA; 11 Neurogenetics of Vocal Communication Group, Max Planck Institute for Psycholinguistics, Nijmegen, the Netherlands; 12 Department of Ecology and Evolution, University of Lausanne, Lausanne, Switzerland; 13 Museum für Naturkunde, Leibniz-Institute for Evolution and Biodiversity Science, Berlin, Germany; 14 School of Biological Sciences, University of Bristol, Bristol, UK; 15 Department Biology II, Ludwig Maximilians University Munich, München, Martinsried Germany; 16 Department of Biology, State University of New York, Geneseo, NY USA; 17 Instituto de Ecología, Universidad Nacional Autónoma de México, Ciudad Universitaria, Mexico City, Mexico; 18 Lubee Bat Conservancy, Gainesville, FL USA; 19 School of Biology and Environmental Science, University College Dublin, Belfield, Dublin 4 Ireland; 20 Donders Institute for Brain, Cognition and Behaviour, Nijmegen, the Netherlands; 21 School of Biology, The University of St Andrews, Fife, UK; 22 Department of Psychology, Neuroscience and Behaviour, McMaster University, Hamilton, ON Canada; 23 Estación de Biología de Chamela, Instituto de Biología, Universidad Nacional Autónoma de México, San Patricio, Mexico; 24 Departamento de Zoología, Instituto de Biología, Universidad Nacional Autónoma de México, Ciudad de México, Mexico

**Keywords:** Methylation analysis, Epigenomics, Ageing

Correction to: *Nature Communications* 10.1038/s41467-021-21900-2, published online 12 March 2021

The previous version of the Supplementary Information associated with this Article omitted Supplementary Figures 1–6. The HTML has been updated to include a corrected version of the Supplementary Information.

## Supplementary information

Updated Supplementary information

