# Supplementary material for: DNA methylation predicts age and provides insight into exceptional longevity of bats
Source: Nat Commun. 2021 Mar 12;12:1615. doi: 10.1038/s41467-021-21900-2 (PMC7955057; doi:10.1038/s41467-021-21900-2)
Supplement: Supplementary file 3 — Reporting Summary [file 41467_2021_21900_MOESM3_ESM.pdf]

nature research

Corresponding author(s): Gerald S. Wilkinson
Steve Horvath

Last updated by author(s): Feb 1, 2021

Reporting Summary

Nature Research wishes to improve the reproducibility of the work that we publish. This form provides structure for consistency and transparency in reporting. For further information on Nature Research policies, see our Editorial Policies and the Editorial Policy Checklist.

Statistics

For all statistical analyses, confirm that the following items are present in the figure legend, table legend, main text, or Methods section.

n/a	Confirmed	
☐	☒	The exact sample size (*n*) for each experimental group/condition, given as a discrete number and unit of measurement
☐	☒	A statement on whether measurements were taken from distinct samples or whether the same sample was measured repeatedly
☐	☒	The statistical test(s) used AND whether they are one- or two-sided<br>*Only common tests should be described solely by name; describe more complex techniques in the Methods section.*
☐	☒	A description of all covariates tested
☐	☒	A description of any assumptions or corrections, such as tests of normality and adjustment for multiple comparisons
☐	☒	A full description of the statistical parameters including central tendency (e.g. means) or other basic estimates (e.g. regression coefficient) AND variation (e.g. standard deviation) or associated estimates of uncertainty (e.g. confidence intervals)
☐	☒	For null hypothesis testing, the test statistic (e.g. *F*, *t*, *r*) with confidence intervals, effect sizes, degrees of freedom and *P* value noted<br>*Give P values as exact values whenever suitable.*
☒	☐	For Bayesian analysis, information on the choice of priors and Markov chain Monte Carlo settings
☒	☐	For hierarchical and complex designs, identification of the appropriate level for tests and full reporting of outcomes
☐	☒	Estimates of effect sizes (e.g. Cohen's *d*, Pearson's *r*), indicating how they were calculated

Our web collection on statistics for biologists contains articles on many of the points above.

Software and code

Policy information about availability of computer code

Data collection	MEME Suite_FIMO_4.12.0: Find Individual Motif Occurrences in a set of probe sequences<br>R_4.0.2: Programming language for statistical computing<br>R_Chipseeker_1.8.6: Determine distance of CpG to nearest transcription start site<br>R_QuasR_1.30.0: Align probe sequences to genome assembly<br>R_sesame_1.3.0: Normalize Illumina Infinium DNA methylation array data
Data analysis	BART_2.0: Predicts functional factors that bind at cis-regulatory regions to regulate gene expression<br>Bedtools_2.25.0: Evaluate overlap between probe sets and transcription factor motifs on array<br>eFORGE_2.0: Functional overlap analysis to identify tissue-specific signal for a set of EWAS DMPs<br>JMP Pro_14.1: Predictive analytics software for statistical analysis and visualization<br>METAL_03-25-11: Facilitates meta-analysis of large datasets in a memory efficient manner<br>PANTHER_16: Gene Ontology enrichment analysis<br>R_4.0.2: Programming language for statistical computing<br>R_glmnet_4.0-2: Fits lasso or elastic-net generalized linear models<br>R_nlme_3.1-151: Fits linear and nonlinear mixed effect models<br>R_phyper_: Probability for the hypergeometric distribution<br>R_samtools_2.6.0: Utilities for manipulating sequence alignments<br>R_WGCNA_1.69: Weighted correlation network analysis

For manuscripts utilizing custom algorithms or software that are central to the research but not yet described in published literature, software must be made available to editors and reviewers. We strongly encourage code deposition in a community repository (e.g. GitHub). See the Nature Research guidelines for submitting code & software for further information.

Data

Policy information about availability of data

All manuscripts must include a data availability statement. This statement should provide the following information, where applicable:

- Accession codes, unique identifiers, or web links for publicly available datasets
- A list of figures that have associated raw data
- A description of any restrictions on data availability

All data used in this study are freely available. Normalized methylation values for each sample, along with sample metadata, are available from NCBI GEO as series GSE164127 (https://www.ncbi.nlm.nih.gov/geo/query/acc.cgi?acc=GSE164127). The design of the Illumina microarray (HorvathMammalMethylChip40) is available from the Gene Expression Omnibus (GEO) at NCBI as platform GPL28271 (https://www.ncbi.nlm.nih.gov/geo/query/acc.cgi?acc=GPL28271). Microarray probe annotations for ten bat genomes are available from the Digital Repository at the University of Maryland (DRUM) at http://hdl.handle.net/1903/26373. Coefficients from the penalized regressions used to estimate bat age for different taxonomic groups are available at https://doi.org/10.6084/m9.figshare.c.5257271. Transcription factor databases used in this study are available as follows: TRANSFAC (http://gene-regulation.com/pub/databases.html), UniPROBE (http://thebrain.bwh.harvard.edu/uniprobe/), HT-Selex (https://ccg.epfl.ch/htpselex/) and JASPAR (http://jaspar.genereg.net/downloads/). Source data for figures are provided with this paper.

Field-specific reporting

Please select the one below that is the best fit for your research. If you are not sure, read the appropriate sections before making your selection.

☐ Life sciences ☐ Behavioural & social sciences ☒ Ecological, evolutionary & environmental sciences

For a reference copy of the document with all sections, see nature.com/documents/nr-reporting-summary-flat.pdf

Ecological, evolutionary & environmental sciences study design

All studies must disclose on these points even when the disclosure is negative.

Study description

This study presents results from analyses of DNA methylation obtained from 712 unique samples of wing tissue DNA from 26 different species of bats of known age. Each sample was assayed with a custom microarray that measures relative methylation at 37,492 sites. Three sets of analyses are presented. The first utilizes elastic net regression to predict age from a subset of sites, i.e. create an epigenetic clock. We illustrate the reliability of the clock using two cross-validation schemes, one which leaves one sample out, and another which leaves one species out, while estimating age from the remaining samples. The second analysis compares the rate of methylation change using a common set of 2000 age-associated sites against the maximum recorded lifespan for each of 23 species for which we had 10 or more samples using a linear model which controls for phylogenetic topology and branch lengths. The final set of analyses involve several enrichment tests to determine if age or longevity sites or the genes they are near are distributed nonrandomly in bat genomes with respect to genomic regions or predicted transcriptional regulators. We identified longevity sites as those with a significant interaction (after BY multiple testing correction) between age and longevity class using data for 5 species from two longevity classes: three long-lived species (Desmodus rotundus, Myotis myotis, and Rhinolophus ferrumequinum) and two short-lived species (Molossus molossus and Leptonycteris curasoae). We identified age-associated sites as the 2000 most highly significant sites from a meta-analysis of methylation versus age correlations for the 19 species with 15 or more samples.

Research sample

To capture the age-related variation in DNA methylation profiles among bats, we obtained wing tissue samples from known-age individuals from 26 different bat species (listed below). We selected this set of species based on their representation of the major families within the order Chiroptera, the availability of a recorded maximum lifespan in the AnAge online database (https://genomics.senescence.info/species/), and the availability of tissue samples from known-age individuals. We obtained tissue samples from colleagues or coauthors who are either involved in long-term field work on marked individuals or keep captive populations of bats. Although we aimed to have samples of both sexes for each species, this was not possible for all species due to the nature of the long-term studies and limited sample availability. The following comma-delimited table, which also appears as Table 1 in the manuscript, provides a summary of the samples for each species. Information includes: Source (F=field-caught, C=captive), number of females (NF), number of males (NM), number of individuals with known exact ages (Exact), age of youngest sample (Yg), age of oldest sample (Old), and the maximum recorded age for that species (Max age). Capture locations for field populations are listed below in the Timing and Spatial Scale. Original capture locations for most captive populations is unknown.

Genus species (Family),Source,NF,NM,Exact,Yg,Old,Max age
Antrozous pallidus (V),F,21,2,1,0.2,7,14.8
Artibeus jamaicensis (Ph),C,3,0,2,11,13,19.2
Carollia perspicillata (Ph),C,17,15,32,0.2,10.5,17.0
Cynopterus brachyotis (Pt),C,6,4,10,6.7,12.9,13.0
Desmodus rotundus (Ph),C,27,17,41,0.3,17.3,29.9
Eidolon helvum (Pt),C,17,7,24,3.4,16.5,21.8
Eptesicus fuscus (V),C,18,41,59,0.3,18.3,23.0
Leptonycteris yerbabuenae (Ph),F,5,6,7,0.2,5,10.1
Molossus molossus (M),F,9,5,6,0.3,5.9,5.9
Myotis lucifugus (V),F,11,0,1,0.1,5,34.0
Myotis myotis (V),F,36,2,33,1,9,37.1
Myotis vivesi (V),F,11,6,4,0.1,2,10.0
Nyctalus noctula,F,3,0,2,0.9,2,12.0

Phyllostomus discolor (Ph),C,31,19,42,0.1,17.7,18.0
Phyllostomus hastatus (Ph),F,61,10,52,0.1,16.5,22.0
Pteropus giganteus (Pt),C,0,4,4,10.9,14.2,44.0
Pteropus hypomelanus (Pt),C,28,12,40,0.4,19.3,26.5
Pteropus poliocephalus (Pt),C,10,6,16,6.1,16.7,23.6
Pteropus pumilus (Pt),C,24,22,45,0.8,17.3,17.3
Pteropus rodricensis (Pt),C,12,7,19,4,20.9,28.0
Pteropus vampyrus (Pt),C,27,24,51,0.6,22.4,24.0
Rhinolophus ferrumequinum (R),F,40,0,39,0.1,21.1,30.5
Rhynchonycteris naso (E),F,6,16,15,0.1,6,8.5
Rousettus aegyptiacus (Pt),C,8,8,3,5,14,22.9
Saccopteryx bilineata (E),F,20,9,24,0.2,8.3,11.0
Tadarida brasiliensis (M),C,9,10,15,0.2,6.2,12.0

Sampling strategy

Because of the long-lifespan of bats and the difficulty of obtaining samples from known-age individuals, our sampling strategy was largely opportunistic. However, in cases where more than 50 samples from known-age individuals were available we attempted to include replicate samples at each age spanning the known lifespan of the species as much as possible. For some species we included samples from individuals that were initially banded as adults but then recaptured and sampled many years later. These samples were scored as minimum, rather than exact, age estimates. The number of minimum vs exact age estimates is also indicated in Table 1.

Data collection

DNA for methylation profiling was extracted from 3 or 4 mm tissue punches taken from wing membranes by numerous individuals (see table below). After bisulfite conversion and labeling of the DNA, methylation profiles were obtained by hybridizing labeled DNA to a custom Illumina methylation array (HorvathMammalMethylChip40) and scanning with an Illumina iScan at the UCLA Neuroscience Genomics Core.

Species;Tissue source Author Initials;DNA isolation Author Initials
Antrozous pallidus; BDA; BDA
Artibeus jamaicensis; BP; DMA
Carollia perspicillata; NJF; DMA
Cynopterus brachyotis; BP; DMA
Desmodus rotundus; GGC; GGC
Eidolon helvum; BP; DMA
Eptesicus fuscus; LNC, AVG, HCB, PAF, LJG; DMA
Leptonycteris yerbabuenae; DZM, RAM; DMA
Molossus molossus; DKND; DMA
Myotis lucifugus; GSW; DMA
Myotis myotis; ECT; MLP
Myotis vivesi; ERH; DMA
Nyctalus noctula; DKND; DMA
Phyllostomus discolor; SCV, EL; EL, PD
Phyllostomus hastatus; GSW, DMA; GSW, DMA
Pteropus giganteus; BP; DMA
Pteropus hypomelanus; BP; DMA
Pteropus poliocephalus; BP; DMA
Pteropus pumilus; BP; DMA
Pteropus rodricensis; BP; DMA
Pteropus vampyrus; BP; DMA
Rhinolophus ferrumequinum; RR, GJ, MLP; MLP
Rhynchonycteris naso; LG, MN, FM; LG, MN, FM
Rousettus aegyptiacus; BP; DMA
Saccopteryx bilineata; MK, MN, FM; MN, FM
Tadarida brasiliensis; A. Loller; DMA

Timing and spatial scale

The table below provides the location and time of sample collection for each species. Samples were collected opportunistically during field studies when previously banded individuals were recaptured.
Species; Location of tissue collection; Time of collection (years); approximate spatial scale (if known) in square km for collection sites.
Antrozous pallidus; "44.94°N, 120.38°W"; 2005-2008; 25
Artibeus jamaicensis; "Gainesville, FL"; 2007;2011; unknown
Carollia perspicillata; "Kerzers FR, Switzerland"; 2018; unknown
Cynopterus brachyotis; "Gainesville, FL"; 2007;2011; unknown
Desmodus rotundus; "Bloomfield Hills, MI; UMD"; 2010-2014; unknown
Eidolon helvum; "Gainesville, FL"; 2006;2007;2011; unknown
Eptesicus fuscus; "Rootstown, OH"; 2018-2019; unknown
Eptesicus fuscus; "Hamilton, ON, Canada"; 2020; unknown
Leptonycteris yerbabuenae; "31°38'51.6"" N, 113°28'53.5"" W"; 2019, 1
Molossus molossus; 09°07’ N 79°41’ W; 2013-2016, 10
Myotis lucifugus; "39°12’N, 76°04’W", 1996, 1
Myotis myotis; "47°35’N, 2°14’W"; 2013-2018, 1

Myotis vivesi; "29°03’N, 113°00’W"; 2015, 2018; 1
Nyctalus noctula; "47.649928° N, 9.186123° E"; 2013-2015, 10
Phyllostomus discolor; "Munich, Germany"; 2018; unknown
Phyllostomus hastatus; "10.4711°N, 61.1958°W"; 1990-2018, 200
Pteropus giganteus; "Gainesville, FL"; 2006;2011; unknown
Pteropus hypomelanus; "Gainesville, FL"; 2007;2009-2011; unknown
Pteropus poliocephalus; "Gainesville, FL"; 2011; unknown
Pteropus pumilus; "Gainesville, FL"; 2006-2013; unknown
Pteropus rodricensis; "Gainesville, FL"; 2006-2014; unknown
Pteropus vampyrus; "Gainesville, FL"; 2006-2017; unknown
Rhinolophus ferrumequinum; "51.7107°N, 2.2777°W"; 2016-2018, 10
Rhynchonycteris naso; "10° 25' N, 84° 00'W"; 2005-2016, 10
Rousettus aegyptiacus; "Gainesville, FL"; 2006;2011-2013; unknown
Saccopteryx bilineata; "10° 53' N, 85° 46' W; 9° 9' N/79° 51' W"; 2005-2016, 10 Costa Rica, 5 Panama
Tadarida brasiliensis; "Weatherford, Texas"; 2019; ; unknown

Data exclusions

We excluded 24 samples that had insufficient DNA to provide reliable methylation values. We discovered that samples with concentrations below 6 ng/µl could not be accurately scored at all sites on the array. We also did not include samples in clock estimates unless the age was exact or we had independent evidence, such as teeth wear, that the minimum age was likely close to the exact age. We excluded 42 samples for this reason.

Reproducibility

We used two cross-validation schemes to assess the reproducibility of the elastic net regression estimate of age from DNA methylation, i.e. the epigenetic clock. Results from these analyses are presented as figure 1a and figure 1b in the manuscript.
We repeated the analysis of the relationship between epigenetic stability and maximum recorded lifespan 10 times using the ranked list of age-associated sites and including different numbers of sites from 5 to 10,000. The qualitative pattern did not change.
We conducted separate enrichment analyses for up to four different species using probes mapped in the genome of each of those species. We present results from one species in the text and from one or more additional species in the Supplementary Figures.
All attempts at replication were successful in that the patterns reported in the ms were obtained.

Randomization

This is an observational study, so randomization is not relevant. However, we attempted to randomize samples with respect to age, sex, and species when filling 96-well plates for methylation profiling.

Blinding

Blinding was not relevant to our study, because this is an observational study and all available data were used.

Did the study involve field work? ☒ Yes ☐ No

Field work, collection and transport

Field conditions

Wing tissue samples were obtained from known-age individuals of 11 species in the wild. Details on how individuals were captured are provided for each of those species in the Supplement. In all cases, bats were captured inside or as they departed from roosting sites, such as caves, attics, or trees. Climatic conditions vary widely among the field sites and weather conditions were not systematically recorded for this study as they are not immediately relevant. Animals in captivity are either kept in outdoor enclosures with temperature-controlled roosting sites in Florida, or in indoor facilities maintained at room temperature.

Location

The table below provides the location and time of sample collection for each species sampled in the field.

Species; Location of tissue collection; Time of collection (years)
Antrozous pallidus; "44.94°N, 120.38°W"; 2005-2008
Leptonycteris yerbabuenae; "31°38'51.6"" N, 113°28'53.5"" W"; 2019
Molossus molossus; 09°07’ N 79°41’ W; 2013-2018
Myotis lucifugus; "39°12’N, 76°04’W"; 1996
Myotis myotis; "47°35’N, 2°14’W"; 2013-2018
Myotis vivesi; "29°03’N, 113°00’W"; "2015, 2018"
Nyctalus noctula; "47.649928° N, 9.186123° E"; 2013-2015
Phyllostomus hastatus; "10.4711°N, 61.1958°W"; 1990-2018
Rhinolophus ferrumequinum; "51.7107°N, 2.2777°W"; 2016-2018
Rhynchonycteris naso; "10° 25' N, 84° 00'W"; 2005-2016
Saccopteryx bilineata; "10° 53' N, 85° 46' W; 9° 9' N/79° 51' W"; 2005-2016

Access & import/export

Below we provide information on permits that were obtained by each investigator to capture, mark and sample wild bats. We imported bat tissues under CDC permits 2018-04-063, 2018-06-009 and 20200205-0437A and APHIS permit 26136. Note that some investigators shared extracted DNA rather than tissue, so no import permits were required.

Antrozous pallidus; bat capture and sampling conducted with permission of the Pine Creek Conservation Area, the Oregon Department of Fish and Wildlife (permit 081-95), and the John Day Fossil Beds National Monument, National Park Service (permit JODA-2005-SCI-0003).
Leptonycteris yerbabuenae; bat capture and sampling conducted under permit SGPA/DGVS/06361/17 issued to RAM by The Ministry of Environment and Natural Resources, Mexico
Molossus molossus; bat capture and sampling conducted under permits SE/A-112-13, SE/A-73-14, SE/A-95-15, and SE/A-32-17 issued to DKD from the Autoridad Nacional del Ambiente in Panama
Myotis lucifugus; bat capture and sampling conducted under permit SCO-30403 issued to GSW from the Maryland Department of

	Natural Resources<br>Myotis myotis; bat capture and sampling conducted in accordance with guidelines and permits delivered by 'Arrêté' by the Préfet du Morbihan and the University College Dublin animal research ethics committee.<br>Myotis vivesi; bat capture and sampling conducted under permits #7668–15 and 2492–17 from Dirección General de Vida Silvestre, and permits #17–16 and 21–17 from Secretaría de Gobernació, Mexico, and imported under PHS permit #2018-06-009.<br>Nyctalus noctula; bat capture and sampling conducted under permit FIBL1/12 approved by the Veterinäramt Thurgau, Switzerland<br>Phyllostomus hastatus; bat capture and sampling conducted under license #000621 from the Forestry, Ministry of Agriculture, Land and Marine Resources, Trinidad and Tobago, and imported under APHIS permit #26136 and PHS permit #2018-04-063.<br>Rhinolophus ferrumequinum; bat capture at the roost with hand nets under Natural England Project Licences 2015-9918-SCI-SCI; 2016-23583-SCI-SCI; 2017-30137-SCI-SCI issued to RR. Sampling conducted under Natural England licenses 2015-11974-SCI-SCI; 2016-25216-SCI-SCI; 2017-31148-SCI-SCI issued to GJ, with tissue biopsy additionally licensed under Home Office Project Licenses (PPL 30/3025 prior to 2018; P307F1428 from 2018 onwards) and Home Office personal licences.<br>Rhynchonycteris naso; bat capture and sampling conducted under permits (permits 022-2005-OFAU, 108-2006-SINAC, 147-2007-SINAC, 183-2008-SINAC, 187-2009-SINAC, 130-2010-SINAC and 068-2011-SINAC, 115–2012-SINAC, 033–2013-SINAC, SINAC-SE-GASP-PI-R-121–2013, R-006–2015-OT-CONAGEBIO, SINAC-SE-CUS-PI-R-088–2016) granted by the MINAE (Ministerio del Ambiente y Energia) and the ACC (Área de Conservación Central) in Costa Rica.<br>Saccopteryx bilineata; bat capture and sampling conducted in Panama was approved by the Smithsonian Tropical Research Institute and its Animal Care and Use Committee (ACUC, permits: IACUC 100316-0910-12, ACUC 2013-1015-2016). For research in Costa Rica, bat capture and sampling permits (272-2003-OFAU, 135-2004-OFAU, 022-2005-OFAU, 108-2006-SINAC, 147-2007-SINAC, 183-2008-SINAC, 187-2009-SINAC, 130-2010-SINAC and 068-2011-SINAC, 115–2012-SINAC, 033–2013-SINAC, SINAC-SE-GASP-PI-R-121–2013, R-006–2015-OT-CONAGEBIO, SINAC-SE-CUS-PI-R-088–2016) were granted by the MINAE (Ministerio del Ambiente y Energia), the ACC (Área de Conservación Central) and the ACG (Área de Conservación Guanacaste).
Disturbance	As the contributors are engaged in long-term field studies, care was taken to minimize disturbance. The presence of marked animals at the same sites over periods in excess of 20 years for several species is evidence that disturbance has been minimized.

Reporting for specific materials, systems and methods

We require information from authors about some types of materials, experimental systems and methods used in many studies. Here, indicate whether each material, system or method listed is relevant to your study. If you are not sure if a list item applies to your research, read the appropriate section before selecting a response.

Materials & experimental systems

n/a	Involved in the study
☒	☐ Antibodies
☒	☐ Eukaryotic cell lines
☒	☐ Palaeontology and archaeology
☐	☒ Animals and other organisms
☒	☐ Human research participants
☒	☐ Clinical data
☒	☐ Dual use research of concern

Methods

n/a	Involved in the study
☒	☐ ChIP-seq
☒	☐ Flow cytometry
☒	☐ MRI-based neuroimaging

Animals and other organisms

Policy information about studies involving animals; ARRIVE guidelines recommended for reporting animal research

Laboratory animals	The study did not involve lab animals
Wild animals	All samples were taken from live, individually marked animals that were subsequently released at the site of capture. No animals were killed. The number of male and female individuals is indicated in Table 1. Additional information on the age and sex of every individual sampled is available in sample metadata as part of the NCBI GEO accession, GSE164127.
Field-collected samples	Wing tissue samples were obtained from known-age individuals of 11 species in the wild. Details on how individuals were captured are provided for each of those species in the Supplement. In all cases, bats were either captured inside or as they departed from roosting sites, such as caves, attics, or trees. Climatic conditions vary widely among the field sites and weather conditions were not systematically recorded for this study as they do not covary with age or with recorded lifespan. Animals kept in captivity are either kept in outdoor enclosures with temperature-controlled roosting sites at the Lubee Bat Conservancy in Florida and McMaster University, or in indoor facilities maintained at room temperature.
Ethics oversight	Institutional animal care and use protocols, or equivalent information from non-US contributors, is provided for each species in the Supplementary methods. Nonuniversity organizations are certified either by the Associated Zoos and Aquariums (Lubee Bat Conservancy) or by the Global Federation of Animal Sanctuaries (Bat World Sanctuary).

Note that full information on the approval of the study protocol must also be provided in the manuscript.
